# An Information-Theoretic Approach to Self-Organisation: Emergence of Complex Interdependencies in Coupled Dynamical Systems

**DOI:** 10.3390/e20100793

**Published:** 2018-10-16

**Authors:** Fernando Rosas, Pedro A.M. Mediano, Martín Ugarte, Henrik J. Jensen

**Affiliations:** 1 Department of Mathematics, Imperial College London, London SW7 2AZ, UK; 2 Centre of Complexity Science, Imperial College London, London SW7 2AZ, UK; 3 Department of Electrical and Electronic Engineering, Imperial College London, London SW7 2AZ, UK; 4 Department of Computing, Imperial College London, London SW7 2AZ, UK; 5 CoDE Department, Université Libre de Bruxelles, B-1050 Brussels, Belgium; 6 Institute of Innovative Research, Tokyo Institute of Technology, Yokohama 226-8502, Japan

**Keywords:** self-organisation, multivariate information theory, coupled dynamical systems, partial information decomposition, statistical synergy, high-order interactions

## Abstract

Self-organisation lies at the core of fundamental but still unresolved scientific questions, and holds the promise of de-centralised paradigms crucial for future technological developments. While self-organising processes have been traditionally explained by the tendency of dynamical systems to evolve towards specific configurations, or attractors, we see self-organisation as a consequence of the interdependencies that those attractors induce. Building on this intuition, in this work we develop a theoretical framework for understanding and quantifying self-organisation based on coupled dynamical systems and multivariate information theory. We propose a metric of global structural strength that identifies when self-organisation appears, and a multi-layered decomposition that explains the emergent structure in terms of redundant and synergistic interdependencies. We illustrate our framework on elementary cellular automata, showing how it can detect and characterise the emergence of complex structures.

## 1. Introduction

### 1.1. Context

It is fascinating how some systems acquire organisation spontaneously, evolving from less to more organised configurations in the absence of centralised control or an external driver. In a world constricted by the second law of thermodynamics and driven by “no free lunch” principles, self-organisation phenomena dazzle us by creating structure seemingly out of nowhere. Besides this aesthetic dimension, self-organisation plays a key role at the core of out-of-equilibrium statistical physics [1], developmental biology [2], and neuroscience [3]. Additionally, self-organisation serves as inspiration for new paradigms of de-centralised organisation where order is established spontaneously without relying on an all-knowning architect or a predefined plan, such as with the Internet of Things [4,5] and blockchain technologies [6]. In this context, self-organisation is regarded as an attractive principle for enabling robustness, adaptability and scalability into the design and managment of large-scale complex networks [7,8,9].

Originally, the notion of self-organisation was introduced in the field of cybernetics [10,11]. These seminal ideas quickly propagated to almost all branches of science, including physics [1,12], biology [2,13], computer science [14,15], language analysis [16,17], network management [18,19], behavioral analysis [20,21] and neuroscience [22,23]. Despite this success, most working definitions of self-organisation still avoid formal definitions and rely on intuitions following an “I know when I see it” logic, which might eventually prevent further systematic developments [24]. Formulating formal definitions of self-organisation is challenging, partly because self-organisation has been used in diverse contexts and with different purposes [25], and partly due to the fact that the basic notions of “self” and “organisation” are already problematic themselves [26].

The absence of an agreed formal definition, combined with the relevance of this notion for scientific and technological advances, generates a need for further explorations about the principles of self-organisation.

### 1.2. Scope of this Work and Contribution

In the spirit of Reference [27], we explore to what extent an information-theoretic perspective can illuminate the inner workings of self-organising processes. Due to the connections between information theory and thermodynamics [28,29], our approach can be seen as an extension of previous works that relate self-organisation and statistical physics (see e.g., [30,31,32]). In previous research, self-organisation has been associated with a reduction in the system’s entropy [30,33,34]—in contrast, we argue that entropy reduction alone is not a robust predictor of self-organisation, and additional metrics are required.

This work establishes a way of understanding self-organising processes that is consistent with the Bayesian interpretation of information theory, as described in Reference [28]. One contribution of our approach is to characterise self-organising processes using multivariate information-theoretic tools—or, put differently, to provide a more fine-grained description of the underlying phenomena behind entropy reduction. We propose that self-organising processes are driven by spontaneous creation of interdependencies, while the reduction of entropy is a mere side effect of this. Following this rationale, we propose the *binding information* [35] as a metric of the strength of the interdependencies in out-of-equilibrium dynamical systems.

Another contribution of our framework is to propose a multi-layered metric of organisation, which combines quantitative and qualitative aspects. Most proposed metrics of organisation in the field of complex systems try to map the whole richness of possible structures into a single dimension [36]. In contrast, drawing inspiration from theoretical neuroscience [37,38], we put forward a multi-dimensional framework that allows for a finer and more subtle taxonomy of self-organising systems. Our framework builds on ideas based on the *Partial Information Decomposition* (PID) framework [39], which distinguishes various information sharing modes in which the binding information is distributed accross the system. This fundamental distinction overcomes counterintuitive issues of existent multiscale metrics for structural complexity, such as the one reported in References [40,41], including negative information values that do not have operational meaning.

A final contribution of this work is to establish a novel connection between information theory and dynamical systems. The standard bridge between these two disciplines includes symbolic dynamics, the Kolmogorov-Sinai entropy, Rényi dimensions and related concepts [42]. In contrast, in this paper we propose to apply information-theoretic analyses over the statistics induced by invariant measures over the attractors. In this way, attractors can be seen as statistical structures that generate interdependencies between the system’s coordinates. This statistical perspective enriches standard analyses of atractors based on fractal dimensions and other geometrical concepts.

The rest of this paper is structured as follows. First, Section 2 briefly introduces the key ideas of this work. Then, Section 3 discusses fundamental aspects of the definition of self-organisation and coupled dynamical systems. Section 4 presents the core ideas our information-theoretic approach, which are then developed quantitavely in Section 5. Our framework is illustrated in Section 6 with an application to elementary cellular automata. Finally, Section 7 discusses our findings and summarises our main conclusions.

## 2. Key Intuitions

This section introduces the key ideas of our framework in an intuitive fashion. These ideas are made rigorous in the following sections.

### 2.1. The Marble Sculpture Analogy

**Figure 1:**
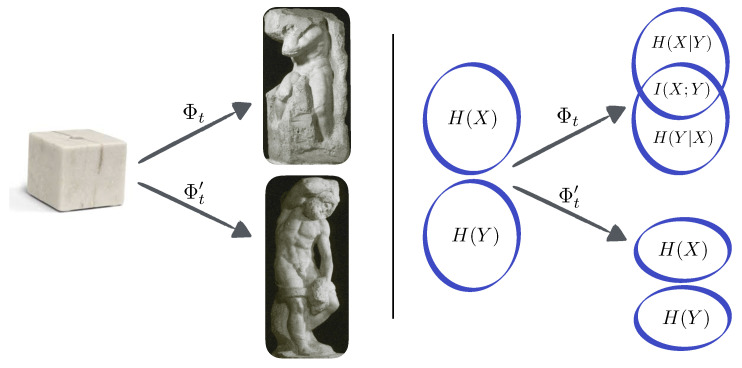
(**Left**) Two maps correponding to two dynamical systems, denoted by Φ and $\Phi^{\prime }$, seen as a sculptor who takes away “superfluous” entropy/marble to let structures appear from inside. The figure shows *The Atlas* and *The Bearded Slave* (circa 1525–30) by Michelangelo Buonarroti, who was famous for letting his figures emerge from the marble “as though surfacing from a pool of water” [43] (pictures taken from commons.wikimedia.org). (**Right**) Likewise, the joint entropy of two (or more) agents could decrease either because they become less random individually ($\Phi^{\prime }$), or because they become correlated (Φ). In this article we provide tools to measure how self-organising systems shape distributions as entropy is reduced—or marbled is carved out—from the initial state.

The overall configuration of a group of agents can be represented by a probability distribution over the set of their possible configurations. Independent agents who are maximally random are characterised by “flat” distributions (technically, distributions that satisfy the *maximum entropy principle* [28]). The temporal evolution of the system then “shapes” this distribution, in the same way as a sculptor shapes a flat piece of marble into a sculpture (c.f. Figure 1). In our view, the shape of the resulting distribution encodes the key properties that emerge from the temporal dynamics of the system, and a substantial part of our framework is to provide tools to measure and describe various types of sculptures. Importantly, just as the sculptor reveals a figure by removing the superfluous marble that is covering it, the temporal evolution generates interdependencies not by adding anything but by reducing the randomness/entropy of the system.

### 2.2. Self-Organisation Versus Dissipation

Consider two agents with random and uncorrelated initial states, as in the analogy above. Their joint entropy, which quantifies their collective randomness, can be depicted as two circles, the size of each circle being proportional to how random the corresponding agent is (c.f. Figure 1). The circles are shown disjoint to reflect the fact that the agents are initially uncorrelated. From this initial situation, there are two qualitatively different ways in which their joint entropy can decrease: the state of each agent could become less random in time, while their independency is preserved; or the agents could become correlated while their individual randomness is preserved. Although both cases show overall entropy reduction, one needs to distinguish finer features of the shape of the resulting distribution to discriminate between genuine self-organisation in the latter scenario and mere dissipation in the former.

## 3. The Goal and Constraints of Self-Organisation

Which dynamical properties enable agents to self-organise? Beyond superficial differences, most studies agree that proper self-organisation requires three fundamental principles to hold:

(i) **Global structure:** the system evolves from less to more structured collective configurations.
(ii) **Autonomy:** agents evolve in the absence of external guidance.
(iii) **Horizontality:** no single agent can determine the evolution of a large number of other agents.

The principles of autonomy and horizontality constitute constraints, in the sense that a system that is not autonomous or horizontal cannot be called *self*-organising. Conversely, the principle of global structure is closer to a goal to be achieved. Hence, one could reformulate the above definition of self-organisation as the following optimisation problem:

(1) $$\begin{array}{cc} \mathrm{generate} & \mathrm{Global}\;\mathrm{structure}\\ \mathrm{subject}\mathrm{to} & \mathrm{Autonomy}\;\mathrm{and}\;\mathrm{horizontality}. \end{array}$$

The following subsections provide a formalisation of these three fundamental principles.

### 3.1. Multiple Agents as a Coupled Dynamical System

An elegant way to formalise these ideas is provided by the literature of coupled dynamical systems. Loosely speaking, a dynamical system is a process that evolves in time, such that its present configuration determines its future evolution following a deterministic rule [44]. Differential equations and finite difference equations are examples of dynamical systems. Furthermore, a collection of dynamical systems are said to be coupled if the future state of each process is affected not only by its own state but also by the state of other processes.

Let us consider a system composed by *N* parts or subsystems, which we call “agents” adopting the terminology from the robotics and multi-agent systems literature. However, these agents could correspond to different coordinates of the spatial movement of a single entity  [45], or to sub-systems of heterogenous nature. The set of possible states for the *k*-th agent is denoted as $\Omega_{k}$, and hence the set of possible configurations of the system is $\Omega :=\prod_{k=1}^{N}\Omega_{k}$, henceforth called “phase space.” The configuration of the system at time t∈T⊂[0,∞) is determined by the vector $x_{t}=\left(x_{t}^{1},\cdots ,x_{t}^{N}\right)\in \Omega$, where $x_{t}^{k}\in \Omega_{k}$ is the corresponding state of the *k*-th agent and *T* is a collection of time indices. By assuming that the agents constitute coupled dynamical systems, the evolution of the group of agents is determined by a collection of maps $\left\{\phi_{t}^{\left(h\right)}\right\}$ with h≥0, where $\phi_{t}^{\left(h\right)}:\Omega \to \Omega$ drives the evolution of the system such that $x_{h+t}=\phi_{t}^{\left(h\right)}\left(x_{h}\right)$. Intuitively, *h* corresponds to an initial time and *t* is the length of the evolution process.

Please note that the choice of deterministic coupled dynamical systems as the basis of our framework has been made for simplicity of presentation. The generalisation of our ideas and methods to stochastic dynamics is straightforward.

### 3.2. Formalising Self-Organisation

We now discuss aspects of the formalisation of (1) based on the language of dynamical systems.

#### 3.2.1. Autonomy

Intuitively, we say that a system is autonomous if it has no architect or “mastermind” controlling its evolution from the outside. Using the dynamical systems language introduced above, we can readily define necessary conditions for the autonomy of a system: we say that a system is autonomous if the collection of maps $\left\{\phi_{t}^{\left(h\right)}\right\}$ are time-invariant—i.e., if its temporal evolution looks the same independently of the initial time *h*. Technically, autonomy requires that $\phi_{t}^{\left(h_{1}\right)}\left(x_{h_{1}}\right)=\phi_{t}^{\left(h_{2}\right)}\left(x_{h_{2}}\right)$ for any $h_{1},h_{2}\in T$ and $x_{h_{1}}=x_{h_{2}}$. This symmetry ensures that there is no organising influence guiding the system from outside. In the rest of this manuscript time translation symmetry is assumed, which allows us to disregard the starting time and drop the superscript (h), using $\phi_{t}$ as a shorthand notation [46].

#### 3.2.2. Horizontality and Locality

Intuitively, horizontality implies a similar restriction within the system itself, in the sense that no small set of units should control or influence the behaviour of the rest of the system. However, in contrast with the simplicity with which autonomy can be addressed, the formalisation of horizontality is substantially more challenging. Our approach here is to take a stronger condition than horizontality, namely:

(iii-b) **Locality:** agents can only interact with a small number of other agents.

Locality is a sufficient condition for horizontality, since if no agent can interact with many other agents then the direct influence of each agent is limited. Conveniently, locality can be elegantly addressed within the framework of coupled dynamical systems. To do this, let us first introduce the notation $\phi_{t}^{k}$ for the *k*-th coordinate of the map $\phi_{t}$, i.e., $\phi_{t}\left(x\right)=\left(\phi_{t}^{1}\left(x\right),\cdots ,\phi_{t}^{N}\left(x\right)\right)$. Then, one can define the interaction network between agents as follows: there exists a link from agent *i* to agent *j* if $\phi_{t}^{j}\left(x\right)$ is affected by changes in the values of $x_{i}$, the *i*-th coordinate of x.

These directed networks can be encoded by an N×N adjacency matrix $A=[a_{ij}]$, where $a_{ij}=1$ if the *i*-th agent is connected with the *j*-th agent and zero otherwise. Locality is, hence, equivalent to A having sparse rows, imposing a fixed bound restricting the number of non-zero entries in each row.

In the following we assume locality, and leave the formalisation of horizontality for future work.

#### 3.2.3. Structure

One of the biggest challenges in the formalisation of self-organisation is to address the notion of *structure*. A large portion of the literature employs this concept without developing a formal definition of it, relying only on intuitive understanding. Furthermore, authors from different fields point towards this same intuition using related but different concepts, including global behaviour, organisation, coordination, or pattern.

Existing approaches to attempt a formalisation of the notion of structure use either attractors, or minimal description length and Kolmogorov complexity. These approaches, and their drawbacks, are discussed in Appendix A. Our own approach, which relies in multivariate information theory, is presented in the next Section.

## 4. Structure As Multi-Layered Statistical Interdependency

This section introduces our framework to study emergence of structure in coupled dynamical systems. The key idea in our approach is to understand *structure as statistical interdependency* and, hence, to regard patterns as deviations from statistical independence, i.e., as interdependent random variables. As argued below, these statistical interdependencies are best described using tools from multivarate information theory.

Adopting an information-theoretic perspective requires a step of abstraction, namely to place the analysis not in trajectories but in ensembles, as explained in Section 4.1. Then, Section 4.2 explores the relationship between the dynamics of the joint Shannon entropy and the increase of statistical interdependency. This discussion is further developed by introducing a decomposition of the Shannon entropy in Section 4.3 and Section 4.4.

For simplicity of exposition, in the rest of the paper we will focus in the case of discrete phase space Ω. However, most of our results still hold for continuous dissipative systems.

### 4.1. From Trajectories to Stochastic Processes

Traditionally, the study of dynamical systems is fundamentally built on how individual trajectories explore the space of possible system configurations. In fact, the measure-theoretic objects that are more studied within dynamical system theory (namely, invariant measures [47]) are distributions that are derived from mean values over trajectories. However, the information-theoretic perspective works not over trajectories but over ensembles (i.e., probability distributions). Associating entropy values to trajectories is usually problematic, as it involves a number of ad-hoc —and often unacknowledged— assumptions (see Endnote [48]—which refers to Reference [49,50]). We make our assumptions explicit, and develop our analysis on an ensemble of systems initialised with stochastic initial conditions. The technicalities behind this approach are developed in the sequel.

Let us consider the case where the initial condition of the system is not a particular configuration $x_{0}\in \Omega$, but an ensemble of configurations described by a probability distribution $\mu_{0}$. Interestingly, the map $\phi_{t}$ not only induces a dynamic on the space of configurations Ω, but it also induces a dynamic on the space of all probability distributions over Ω, denoted as M(Ω). Consider, as an example, the discrete distribution $\mu_{0}=\sum_{j=1}^{\infty }c_{j}\delta_{x_{j}}$ where $\delta_{x_{j}}$ is the Dirac delta (or the Kronecker delta if Ω is discrete). For this measure, the probability of a subset of configurations O⊂Ω is calculated by $\mu_{0}\left(O\right)=\sum_{j=1}^{\infty }c_{j}1_{x_{j}}\left(O\right)$, where $1_{x_{j}}\left(O\right)=1$ if $x_{j}\in O$ and zero otherwise. A natural time-evolution of this probability distribution is given by $\mu_{t}=\sum_{j=1}^{\infty }c_{j}1_{\phi_{t}\left(x_{j}\right)}$. One can generalise this construction for an arbitrary initial probability distribution $\mu_{0}$ by introducing the Frobenius-Perron operator [51], which is an operator over M(Ω) defined as

(2) $$\Phi_{t}\left\{\mu_{0}\right\}\left(O\right)\mu_{0}\left(\phi_{t}^{-1}\left(O\right)\right)=\mu_{0}\left(\{x\in \Omega |\phi_{t}\left(x\right)\in O\}\right)\;.$$

Note that the collection $\left\{\Phi_{t}\left\{\cdot \right\},t\in T\right\}$ generates a dynamic over M(Ω), and hence constitutes a new dynamical system (see Endnote [52]—which refers to Reference [53]).

The set of probability distributions $\left\{\mu_{t}=\Phi_{t}\left\{\mu_{0}\right\},t\in T\right\}$ induces a corresponding multivariate stochastic process $X_{t}=\left(X_{t}^{1},\cdots ,X_{t}^{N}\right)=\phi_{t}\left(X_{0}\right)$, which follows a joint probability distribution $p_{X_{t}}=\mu_{t}$ (for the complete statistics of $X_{t}$ and technical details of this correspondence, see Appendix B). Note that the properties of this stochastic process are completely determined by the initial distribution $\mu_{0}$ and the map $\phi_{t}$. Each sub-process $X_{t}^{k}$ describes the uncertainty related to the state of the agent *k* at time *t*, the statistics of which are found by marginalising the joint statistics of $p_{X_{t}}$. The aim of the next subsections is to explore the statistiscal interdependencies that can exist among these sub-processes.

### 4.2. Information Dynamics

The joint Shannon entropy of the system at time *t*, given by $H\left(X_{t}\right)-\sum_{x\in \Omega }p_{X_{t}}\left(x\right)\log p_{X_{t}}\left(x\right)$, corresponds to the information required to resolve the uncertainty about the state of the system at time *t* (see Appendix C). The uncertainty reflected by this entropy has two sources [54]. One source is stochasticity in the initial condition, i.e., when the initial configuration of the system at time t=0 is not fully determined, but only prescribed by a probability distribution. The second source of uncertainty are stochastic dynamics (also known as “dynamical noise”), i.e., when the time evolution could make the system potentially transit from a single starting configuration to two or more different future configurations. Dynamical systems have deterministic transitions, and hence only exhibit the first type of uncertainty.

When considering discrete phase spaces, the deterministic dynamics guarantee that the uncertainty due to random initial conditions cannot increase; it can only decrease or be conserved. As a simple example, let us consider a dynamical system with a single point attractor: even if one does not know where a trajectory starts, one knows that the trajectory ends in the attracting point. In this case, any information encoded in the initial condition is vanished by the dynamics, as one cannot find out where trajectories are coming from. We call this phenomenon “information dissipation,” which mathematically can be stated as:

(3) $$H\left(X_{t}\right)\geq H\left(X_{t+h}\right)\;\mathrm{for}\;\mathrm{all}\;\;h>0\;.$$

Due to the deterministic nature of the time evolution, Equation (3) is guaranteed by the data-processing inequality [55]. This decrease in entropy is not in contradiction with the second law of thermodynamics, as these systems are generally open and connected to an environment [53,56]. The equality in Equation (3) is attained by closed systems, being this a direct consequence of the well-known Liouville theorem [57].

Information dissipation is directly related with the action of attractors. For a given attractor *A*, its basin of attraction B(A) is the largest subset of Ω such that $\lim_{t\to \infty }\phi_{t}\left(x\right)=A$ for all x∈B(A). Intuitively, any trajectory starting in B(A) asymptotically runs into *A*. Similarly, the evolution of an initial distribution $\mu_{0}$ supported on B(A) eventually ends up being supported almost only on *A* when *t* is large enough; correspondingly, its Shannon entropy tends to decrease due to the reduced portion of the phase space where the system is confined to dwell. As such, information dissipation (i.e., entropy decreasing due to the action of attractors) is a necessary condition for self-organisation.

It is tempting to postulate entropy reduction as a strong indicator of self-organisation, based on a loose interpretation of entropy as a metric of disorder. However, the relationship between entropy and disorder is problematic, as disorder has different meanings in various contexts and there exists no single widely accepted definition for it. Moreover, entropy reduction is not a sufficient condition for self-organisation [24]. For example, consider a group of uncopled damped oscillators initialised with random initial positions and velocities. This system evolves towards the resting state where all velocities are zero, which is the only point attractor of the system – thereby reducing its entropy to zero. However, one would not want to call this evolution as one that is promoting self-organisation, as the agents are never engaged in any interaction.

### 4.3. Binding and Residual Information

A key idea that emerges from the previous discussion is to relate organisation with agent interdependency. Following this rationale, we propose that self-organisation is related to the increase of interdependency between the agents due to the dynamics. To formalise this intuition, we explore a decomposition of the total entropy in two parts: one that quantifies interaction and one that measures uncorrelated variability.

To introduce the decomposition, let us first consider the following identity:

(4) $$H\left(X_{t}^{j}\right)=I\left(X_{t}^{j}\;;\;X_{t}^{-j}\right)+H\left(X_{t}^{j}|X_{t}^{-j}\right)\;,$$

where we are using the shorthand notation $X_{t}^{-j}=\left(X_{t}^{1},\cdots ,X_{t}^{j-1},X_{t}^{j+1},\cdots ,X_{t}^{N}\right)$, and I(·;·) is the standard Shannon mutual information. This equality states that the entropy of the state of the *j*-th agent, as quantified by $H(X_{t}^{j})$, can be decomposed into a part that is shared with the other agents, $I(X_{t}^{j};X_{t}^{-j})$, and a part that is not, $H(X_{t}^{j}|X_{t}^{-j})$. This intuition is made rigurous by the Slepian-Wolf coding scheme [58,59], which shows that $I(X_{t}^{j};X_{t}^{-j})$ corresponds to information about the *j*-th agent that can be retrieved by measuring other agents, while $H(X_{t}^{j}|X_{t}^{-j})$ is information that can only be retrieved by measuring the *j*-th agent.

Following the above rationale, the total “non-shared information” in the system is nothing more than the sum of the non-shared information of every agent, and corresponds to the *residual entropy* [35]:

(5) $$R\left(X_{t}\right):=\sum_{j=1}^{N}H\left(X_{t}^{j}|X_{t}^{-j}\right)\;.$$

One can verify that the agents are statistically independent at time *t* if and only if $H\left(X_{t}\right)=R\left(X_{t}\right)$. The complement of the residual entropy corresponds to the *binding information* [60], which quantifies the part of the joint Shannon entropy that is shared among two or more agents. This can be computed as

(6) $$B\left(X_{t}\right):=H\left(X_{t}\right)-R\left(X_{t}\right)=H\left(X_{t}\right)-\sum_{j=1}^{N}H\left(X_{t}^{j}|X_{t}^{-j}\right)\;.$$

Note that the above formula corresponds to a multivariate generalisation of the information-theoretic identity I(X;Y)=H(X,Y)−H(X|Y)−H(Y|X), which captures linear and non-linear dependecies that might exist between two or more agents. As such, the binding information is one of several multivariate generalisations of the mutual information, and is the only one known to enable a non-negative decomposition of the joint entropy [61].

In summary, the binding information provides a natural metric of the strength of the statistical interdependencies within a system. In fact, this metric is consistent with the intuition that a faithful metric of organisational richness should be small for systems with maximal or minimal joint entropy (see Reference [62] and references therein). On the one hand, maximal entropy takes place when agents are independent, which implies that $H\left(X_{t}\right)=R\left(X_{t}\right)$ and hence $B(X_{t})=0$ due to the lack of interaction. On the other hand, entropy is minimised in systems that exhibit no diversity, which limits their binding information due to the fact that $B\left(X_{t}\right)\leq H\left(X_{t}\right)=0$.

Interestingly, although the deterministic nature of deterministic dynamical systems constrains $H(X_{t})$ to be non-increasing, both $B(X_{t})$ and $R(X_{t})$ can increase or decrease. In contrast with the entropy, an increase in binding information is an unequivocal sign that statistical structures are being generated within the system by its temporal evolution.

### 4.4. The Anatomy of the Interdependencies

Although the binding information provides an attractive information-theoretic metric of organisation strength, a one-dimensional description is not rich enough to describe the range of phenomena observed in self-organising agents. To obtain a more detailed picture we use the Partial Information Decomposition (PID) framework, which allows us to develop a finer decomposition of the binding information and distinguish between different modes of information sharing. Originally, PID was introduced to study various aspects of information-theoretic inference, which consider a target variable predicted using the information provided by a number of information sources (see References [39,63,64,65] and references therein). A key intuition introduced by these works is to distinguish between various information modes: in particular, *redundant information* corresponds to information about the target variable that can be retrieved from more than one source, and *synergistic information* corresponds to information that becomes available only when two or more sources are accessed simultaneously.

Traditional PID approaches divide the variables between target and sources, having each of them a very different role in the framework. Nevertheless, it is possible to propose symmetric decompositions of the joint Shannon entropy using PID principles that avoid these dialectic labellings [61,66,67]. In this case, the total information encoded in the system’s configuration is decomposed in redundant, unique and synergistic components. Redundancy takes place when measuring a single agent allows the observer to predict the state of other agents. Synergy corresponds to high-order statistical effects that can constrain groups of variables without imposing low-order restrictions. This idea of synergistic information is a generalisation of the well-known fact that random variables can be pairwise independent while being jointly interdependent. The relationship between synergistic information and high-order correlations in the context of statistical physics has been explored in References [61,63].

The work reported in [61] describes a decomposition for the binding information for the case of systems of N=3 agents. In the sequel, we extend these ideas postulating three formal decompositions of the binding information for larger system sizes. Please note that our approach here is not to establish precise formulas for computing the value of the components of these decompositions for arbitrary underlying probability distributions. Instead, in Section 5.1 we provide universal upper and lower bounds for these components, which need to be satisfied irrespective of the chosen functional form. Moreover, these bounds can be used in some cases to determine exact values of the decomposition’s components, as illustrated in Section 6.

#### 4.4.1. Decomposition by Extension of Sharing

Since the binding information is the information shared by two or more agents, it is natural to discriminate exactly how many agents are involved in the sharing. Following this rationale, we propose the following decomposition:

(7) $$B\left(X_{t}\right)=\sum_{n=2}^{N}b^{n}\left(X_{t}\right)\;,$$

where $b^{n}\left(X_{t}\right)$ measures the portion of the binding information that is shared among exactly *n* agents. The index *n* refers to the number of agents that are linked by the corresponding relationship. Therefore, $b^{n}\left(X_{t}\right)$ quantifies the strength of interdependencies that link groups of *n* agents.

To illustrate these ideas, let us explore some simple examples where this decomposition can be computed directly from our desiderata.

**Example** **1.** *Consider two independent Bernoulli random variables U and V with parameter p=0.5 (i.e H(U)=H(V)=1). Then,*

*(i)*  *If $\left(X_{t}^{1},X_{t}^{2},X_{t}^{3}\right)=\left(U,U,U\right)$, then $R(X_{t})=0$ and $B\left(X_{t}\right)=H\left(U\right)$. Furthermore, because of the triple identity $b^{3}\left(X_{t}\right)=H\left(U\right)$, and hence $b^{2}\left(X_{t}\right)=0$.*
*(ii)*  *If $\left(X_{t}^{1},X_{t}^{2},X_{t}^{3}\right)=\left(U,U,V\right)$, then $R\left(X_{t}\right)=H\left(V\right)$ and $B\left(X_{t}\right)=H\left(U\right)$. In this case, $b^{2}\left(X_{t}\right)=H\left(U\right)$ and hence $b^{3}\left(X_{t}\right)=0$.*
*(iii)*  *If $\left(X_{t}^{1},X_{t}^{2},X_{t}^{3}\right)=\left(U,V,U\left(xor\right)V\right)$, then $R(X_{t})=0$ and $B\left(X_{t}\right)=H\left(U\right)+H\left(V\right)$. Furthermore, due to the triple interdepedency $b^{3}\left(X_{t}\right)=H\left(U\right)+H\left(V\right)$, and hence $b^{2}\left(X_{t}\right)=0$.*

#### 4.4.2. Decomposition by Sharing Modes

Following Reference [61], we distinguish between redundant and synergistic information sharing modes. Redundancy, in this context, refers to information that is disclosed as soon as any of the agents who participate in the sharing are measured. Put differently, if agents are engaged in a redundant information sharing mode then measuring one of them allows the observer to make inferences on the states of the others. Conversely, synergistic information sharing takes place when accessing the state of one agent is not enough to obtain predictive power, i.e., to infer the state of the other agents involved. The key element is then how many agents need to be measured in order to obtain information about the other agents. Synergistic relationships require two or more, implying high-order statistical effects.

Based on these ideas, we postulate the following decomposition:

(8) $$b^{n}\left(X_{t}\right)=\sum_{i=1}^{n-1}I_{i}^{n}\left(X_{t}\right)\;,$$

where $I_{i}^{n}\left(X_{t}\right)$ denotes information that is shared between *n* agents, and becomes fully available after accessing i<n of the agents involved in the sharing. In other words, *i* is the smallest number of agents that enables the use of the information that corresponds to $I_{i}^{n}\left(X_{t}\right)$ for predicting the state of the remaining n−i agents. Note the use of upperscripts and lowerscripts differentiate between group sizes and order of the sharing mode. This decomposition introduces a range of (i,n)-interdependencies, where *n* is the extension of the interdependency (how many agents are involved) while *i* is the “degree of synergy.” With this notation, redundancies correspond to i=1-interdependencies, while $I_{i}^{n}\left(t\right)$ for i≥2 are synergies of order *i*.

Based on these ideas, another way of decomposing the binding information is by focusing on the possible *information sharing modes*, i.e., ways in which information can be shared among the agents according to *i*. By combining Equations (7) and (8), one can then present the following decomposition:

(9) $$B\left(X_{t}\right)=\sum_{n=2}^{N}\sum_{i=1}^{n-1}I_{i}^{n}\left(X_{t}\right)=\sum_{i=1}^{N-1}m_{i}\left(X_{t}\right)\;,$$

where $m_{i}\left(X_{t}\right)\sum_{n=i+1}^{N}I_{i}^{n}\left(X_{t}\right)$ corresponds to information sharing modes that are fully accessed when measuring sets of *i* agents. In particular, $m_{1}\left(X_{t}\right)$ collects all the “redundancies” of the system, i.e., sharing modes that are fully accessed by measuring only one of the agents involved in the sharing. Correspondingly, the terms $m_{i}\left(X_{t}\right)$ for i≥2 convey the strength of synergies and high-order effects.

To contrast these ideas with the previous decomposition, we study the same scenarios from Example 1 under this new perspective.

**Example** **2.** *Consider U and V as defined in Example 1. Then,*

(i) *If $\left(X_{t}^{1},X_{t}^{2},X_{t}^{3}\right)=\left(U,U,U\right)$, then $m_{1}\left(X_{t}\right)=H\left(U\right)$, as the information contained in any variable allows to predict the others, while $m_{2}\left(X_{t}\right)=0$.*
(ii) *If $\left(X_{t}^{1},X_{t}^{2},X_{t}^{3}\right)=\left(U,U,V\right)$, then similarly as above $m_{1}\left(X_{t}\right)=H\left(U\right)$ and $m_{2}\left(X_{t}\right)=0$. Both cases are redundancies (same i) of disimilar extension (different n).*
(iii) *If $\left(X_{t}^{1},X_{t}^{2},X_{t}^{3}\right)=\left(U,V,U\left(xor\right)V\right)$, then measuring one agent does not allow any predictions over the others, while by measuring two agents one can predict the third one (for a discussion on the statistical properties of the xor, please see Reference [61], Section 4.2). This implies that $m_{2}\left(X_{t}\right)=H\left(U\right)+H\left(V\right)$, and hence $m_{1}\left(X_{t}\right)=0$.*

## 5. A Quantitative Method to Study Time-Evolving Organisation

In this section we leverage the ideas discussed in Section 4 to develop a method to conduct a quantitative and qualitative analysis of self-organisation in dynamical systems. The goal of this method is twofold: to detect when self-organisation is taking place, and to characterise it as redundancy- or synergy-dominated. For this, Section 5.1 first develops upper and lower bounds for the terms of the decompositions of the binding information presented in Section 4.4. Then, Section 5.2 outlines a protocol of four steps that can be applied in practical scenarios.

### 5.1. Bounds for the Information Decompositions

#### 5.1.1. Upper Bounds for the Decomposition by Extension

Let us define $\alpha_{L}=\left(\alpha_{1},\cdots ,\alpha_{L}\right)$ to be a vector of *L* integer indices with $1\leq \alpha_{1}<\alpha_{2}<\cdots <\alpha_{L}\leq N$, and $B(X_{t}^{\alpha_{L}})$ to be the binding information of the agents that correspond to those indices at time *t*, i.e.,

(10) $$B\left(X_{t}^{\alpha_{L}}\right)=H\left(X_{t}^{\alpha_{L}}\right)-\sum_{j=1}^{L}H\left(X_{t}^{\alpha_{j}}|X_{t}^{\alpha_{1}},\cdots ,X_{t}^{\alpha_{j-1}},X_{t}^{\alpha_{j+1}},\cdots ,X_{t}^{\alpha_{L}}\right)\;,$$

where $X_{t}^{\alpha_{L}}=\left(X_{t}^{\alpha_{1}},\cdots ,X_{t}^{\alpha_{L}}\right)$. Also, let us denote as $I_{L}$ the set of all index vectors $\alpha_{L}$ of length *L*, which correspond to the possible subsets of *L* agents with cardinality |IL|=NL.

Recall that $b^{n}\left(X_{t}\right)$ corresponds to information that is shared exactly by *n* agents, and hence $\sum_{n=2}^{L}b^{n}\left(X_{t}\right)$ is the information shared by *L* or less agents. As $B(X_{t}^{\alpha_{L}})$ corresponds to the information shared between agents $\alpha_{1},\cdots ,\alpha_{L}$ it is clear that for any L∈{2,⋯,N} the following bounds hold:

(11) ∑n=2Lbn(Xt)≤∑αL∈ILB(XtαL)≤NLmaxαL∈ILB(XtαL).

Although these bounds might not be tight, Equation (11) suggests that $\max_{\alpha_{L}\in I_{L}}B_{\alpha_{L}}\left(t\right)$ can be useful for sizing the value of $\sum_{n=2}^{L}b^{n}\left(t\right)$. In particular, if $\max_{\alpha_{L}\in I_{L}}B_{\alpha_{L}}\left(t\right)=0$ then $b^{n}\left(X_{t}\right)=0$ for all n=2,⋯,L, which due to Equation (7) would imply that $B\left(X_{t}\right)=\sum_{n=L+1}^{N}b^{n}\left(X_{t}\right)$.

These bounds are illustrated in the following example.

**Example** **3.** *Consider U and V as defined in Example 1. Let us focus in L=2, and note that for this case $I_{2}=\left\{\left\{1,2\right\},\left\{1,3\right\},\left\{2,3\right\}\right\}$, and hence*

(12) $$\max_{\alpha_{2}\in I_{2}}B\left(X_{t}^{\alpha_{2}}\right)=\max_{i,j\in \{1,2,3\}}I\left(X_{t}^{i};X_{t}^{j}\right)\;and\;\sum_{\alpha_{2}\in I_{2}}B\left(X_{t}^{\alpha_{2}}\right)=\sum_{i=1}^{3}\sum_{j=i+1}^{3}I\left(X_{t}^{i};X_{t}^{j}\right)\;.$$

*Using this, it is direct to find that:*

*(i)*  *If $\left(X_{t}^{1},X_{t}^{2},X_{t}^{3}\right)=\left(U,U,U\right)$, then 32maxα2∈I2B(Xtα2)=∑α2∈I2B(Xtα2)=3H(U), and hence Equation (11) shows that $b^{2}\left(X_{t}\right)\leq 3H\left(U\right)$. This bound is not tight, as $b^{2}\left(X_{t}^{3}\right)=0$ (c.f. Example 1). Also, note that for L=3 one finds that $\max_{\alpha_{3}\in I_{3}}B\left(X_{t}^{\alpha_{3}}\right)=B\left(X_{t}\right)=H\left(U\right)$, showing that the bounds don’t need to be monotonic on L.*
*(ii)*  *If $\left(X_{t}^{1},X_{t}^{2},X_{t}^{3}\right)=\left(U,U,V\right)$, then $\max_{\alpha_{2}\in I_{2}}B\left(X_{t}^{\alpha_{2}}\right)=\sum_{\alpha_{2}\in I_{2}}B\left(X_{t}^{\alpha_{2}}\right)=H\left(U\right)$. This bound is tight, as $b^{2}\left(X_{t}\right)=H\left(U\right)$ (c.f. Example 1).*
*(iii)*  *If $\left(X_{t}^{1},X_{t}^{2},X_{t}^{3}\right)=(U,V,U$(xor)V), then $\max_{\alpha_{2}\in I_{2}}B\left(X_{t}^{\alpha_{2}}\right)=0$, and hence the bounds determine that $b^{2}\left(X_{t}\right)=0$.*

#### 5.1.2. Upper And Lower Bounds for the Decomposition by Sharing Modes

Let us recall that $m_{i}\left(X_{t}\right)$ accounts for the information about other agents that is obtained when measuring groups of *i* agents, but not less. Similarly, $\sum_{i=1}^{L}m_{i}\left(X_{t}\right)$ is the predictability about other agents that is obtained when accessing *L* or less agents. Therefore, one can provide the following bounds valid for any L∈{1,⋯,N−1}:

(13) ψL(t)≤∑i=1Lmi(Xt)≤∑j=1N∑αL∈ILαi≠jI(XtαL;Xtj)≤NN−1LψL(t),

where we have used the shorthand notation

(14) $$\begin{array}{c} \psi_{L}\left(t\right):=\max_{j\in \{1,\cdots ,N\}}\max_{\begin{array}{c} \alpha_{L}\in I_{L}\\ \alpha_{i}\neq j \end{array}}I\left(X_{t}^{\alpha_{L}};X_{t}^{j}\right)\;. \end{array}$$

As in Equation (11), this shows that $\psi_{L}\left(t\right)$ can be used as a proxy for estimating the relevance of $\sum_{i=1}^{L}m_{i}\left(X_{t}\right)$. In particular, if $\psi_{L}\left(t\right)=0$ then $\sum_{i=1}^{L}m_{i}\left(X_{t}\right)=0$. Therefore, by using Equation (9), if $\psi_{L}\left(t\right)=0$ then all the binding information is composed by synergies of order L+1 or more.

The properties of $\psi_{L}\left(t\right)$ can reveal the distribution of sharing modes across the system. First, note that $\psi_{L}\left(t\right)$ is a non-decreasing function of *L*: information (in the Shannon sense) “never hurts,” and hence having larger groups of agents for making predictions cannot reduce predictive power. Secondly, in most scenarios $\psi_{L}\left(t\right)$ is concave: the additional perdictability obtained by including one more agent usually shows diminishing returns as *L* grows. In effect, the most informative agents are normally selected first, and hence for large values of *L* one can just add agents with weak informative power, which can also be redundant with the agents already considered. Accordingly, scenarios where $\psi_{L}\left(t\right)$ as function of *L* is concave are called *redundancy-dominated*. In contrast, scenarios in which $\psi_{L}\left(t\right)$ is convex are called *synergy-dominated*. Intuitively, in synergy-dominated scenarios agents might be uninformative by themselves, but become informative when grouped together. Therefore, a convex $\psi_{L}\left(t\right)$ is a sign of a synergistic system, one that exhibits larger predictability gains when *L* grows.

These ideas and bounds are illustrated in the following example.

**Example** **4.** *Consider again U and V as defined in Example 1. Focusing in L=1, one finds that $\psi_{1}\left(t\right)=\max_{i,j\in \{1,\cdots ,N\}}I\left(X_{t}^{i};X_{t}^{j}\right)$. Therefore, one can find that:*

*(i)*  *If $\left(X_{t}^{1},X_{t}^{2},X_{t}^{3}\right)=\left(U,U,U\right)$, then $\psi_{1}\left(t\right)=H\left(U\right)$. Therefore, the bounds in Equation (13) show that $H\left(U\right)\leq m_{1}\left(X_{t}\right)\leq 3H\left(U\right)$.*
*(ii)*  *If $\left(X_{t}^{1},X_{t}^{2},X_{t}^{3}\right)=\left(U,U,V\right)$, then again $\psi_{1}\left(t\right)=H\left(U\right)$, hence the bounds are the same as above.*
*(iii)*  *If $\left(X_{t}^{1},X_{t}^{2},X_{t}^{3}\right)=(U,V,U$(xor)V) then $\psi_{1}\left(t\right)=0$, which in turn guarantees that $m_{1}\left(X_{t}\right)=0$.*

*By noting that $\psi_{2}\left(t\right)=\max\left\{I\left(X_{t}^{1};X_{t}^{2}X_{t}^{3}\right),I\left(X_{t}^{2};X_{t}^{1}X_{t}^{3}\right),I\left(X_{t}^{3};X_{t}^{1}X_{t}^{2}\right)\right\}$, a direct calculation shows that $\psi_{2}\left(t\right)=H\left(U\right)$ for the three above cases. By considering $\psi_{0}\left(t\right):=0$, one finds that cases (i) and (ii) are redundancy-dominated, while case (iii) is synergy-dominated.*

### 5.2. Protocol to Analyse Self-Organisation in Dynamical Systems

Wrapping up these results, we propose the following definitions for self-organisation. Note that these are aimed at quantifying organisation, while the constraints of “self” are guaranteed by restricting to autonomous maps with sparse interaction matrices (see Section 3.2).

**Definition** **1.** *Consider a coupled dynamical system with autonomous evolution and a bounded number of non-zero elements per row in its interaction matrix. Then, the system is self-organising if $B(X_{t})$ is an increasing function of t. Moreover, the value of $B(X_{t})$ is used as a metric of organisation strength.*

**Definition** **2.** *A self-organising process is said to be synergy-dominated if* $\lim_{t\to \infty }\psi_{L}\left(t\right)$ *is convex as function of L. If* $\lim_{t\to \infty }\psi_{L}\left(t\right)$ *is concave, the process is said to be redundancy-dominated.*

Note that for certain processes $\lim_{t\to \infty }\psi_{L}\left(t\right)$ can exhibit a combination of convex and concave segments, which suggests the coexistence of redundant and synergistic structures at different scales. An example of this is discussed in Section 6.2.4.

Following these definitions, we propose the following protocol for analysing a given dynamical system. The steps are:

(1) Check that the maps satisfy autonomy and locality (Section 3.2).
(2) Consider a random initial condition given by a uniform distribution over the phase space, $\mu_{0}$, and use it to drive the coupled dynamical system. This involves initialising the system in the least biased initial configuration, i.e., with maximally random and independent agents.
(3) Compute the evolution of the probability distribution given by $\mu_{t}=\Phi_{t}\left\{\mu_{0}\right\}$. This can be done directly using the map, a master equation [68], or in the case of a finite phase space by computing numerically all the trajectories.
(4) Compute the joint Shannon entropy $H(X_{t})$, the residual information $R(X_{t})$, and the binding information $B(X_{t})$ as a function of *t*.
(5) For values of $t_{0}$ at which $B(X_{t_{0}})>0$, compute $\psi_{L}\left(t_{0}\right)$ for L=1,⋯,N.

Note that by considering a flat initial condition in step (1), one ensures that the system initially has no correlations, i.e., $B(X_{0})=0$. Therefore, if one finds that $B(X_{t})>0$ for some t>0, one can be sure that these interdependencies were entirely created by the dynamics of the system. Also, while step (3) clarifies if self-organisation is taking place following Definition 1, (i.e., by checking if $B(X_{t})>0$ for some t>0), step (4) discriminates between redundant and synergistic organisation structures according to Definition 2.

## 6. Proof of Concept: Cellular Automata

Cellular Automata (CA) are a well-known class of discrete coupled dynamical systems widely used in the study of complex systems and distributed computation [69]. A CA is a multi-agent system in which every agent has a finite set of possible states, and evolves in discrete time steps following a set of simple rules based on its own and other agents’ states. For simplicity, we focus our analysis on synchronous update CA (see Endnote [70]—which refers to Reference [71]).

CA are a natural candidate for our measures, since they have been often used in other studies of self-organisation [72], some of them are capable of universal computation [73], and they provide a rich testbed for theories of distributed computation and collective behaviour in complex systems [74].

### 6.1. Method Description

Our analysis focuses on Elementary Cellular Automata (ECA), which constitute a particular subclass of CA. In ECA, agents (or *cells*) are arranged in a one-dimensional cyclic array (or *tape*). The state of each cell at a given time step has two possible states, 0 or 1, and is a boolean function of the state of itself and its immediate neighbours at the previous time step. The same boolean function dictates the time evolution of all agents, inducing a spatial translation symmetry. Hence, each of the 256 different boolean functions of three binary inputs induces a different *evolution rule*. Rules are then enumerated from 0 to 255 and each ECA, irrespective of its number of agents, can be classified by its rule. Moreover, each rule has an equivalent class of rules, given by the rules obtained by reflection (exchanging right and left) and inversion (exchanging zeros and ones). Keep in mind that all the statistical results discussed in this section are equally valid for all the members of the corresponding equivalence class. For a more detailed description of ECA and their numbering system, see Reference [72].

In our simulations, we followed the protocol outlined in Section 5.2 over arrays of *N* cells that followed one ECA rule. We initialised one copy of the ECA in each of the $2^{N}$ possible initial conditions and numerically computed the temporal evolution of each one of them. As is standard in the ECA literature, the automata were simulated under periodic boundary conditions. The probability distribution at time *t*, $\mu_{t}$, was calculated after the system reached a *pseudo-stationary regime*, which plays the role of a non-equilibrium steady-state [75,76]. These calculations were performed using methods outlined in Appendix D, which allowed us to consider arrays up to size N=17.

Our analysis of the ECA included the following elements:

(a) The temporal evolution of $H(X_{t})$, $B(X_{t})$ and $R(X_{t})$. These plots show if the ECA shows signs of self-organisation according to Definition 1, and if the joint entropy decreases or remains constant (c.f. Section 4.2).
(b) The interdependency between individual cells through time, as given by the mutual information between a single cell at time t=0 and all other cells in the same and successive times (i.e., $I(X_{0}^{0};X_{t}^{k})$ for t∈{0,1⋯} and k∈{1,⋯,N}). This reflects the predictive power of the state of a cell in the initial condition over the future evolution of the system [77].
(c) The mutual information between every pair of cells for the pseudo-stationary distribution. Because of the spatial translation symmetry of ECA, it suffices to take any cell and compute its mutual information with each other cell. We call this “spatial correlation,” as it measures interdependencies between cells at the same time *t*.
(e) The curve $\psi_{L}$ (c.f. Section 5) for the pseudo-stationary distribution, which is used to characterise a self-organising system as either redundancy- or synergy-dominated as per Definition 2. This curve can also be interpreted as how much of a cell can be predicted by the most informative group of *L* other cells.

### 6.2. Results

**Figure 2:**
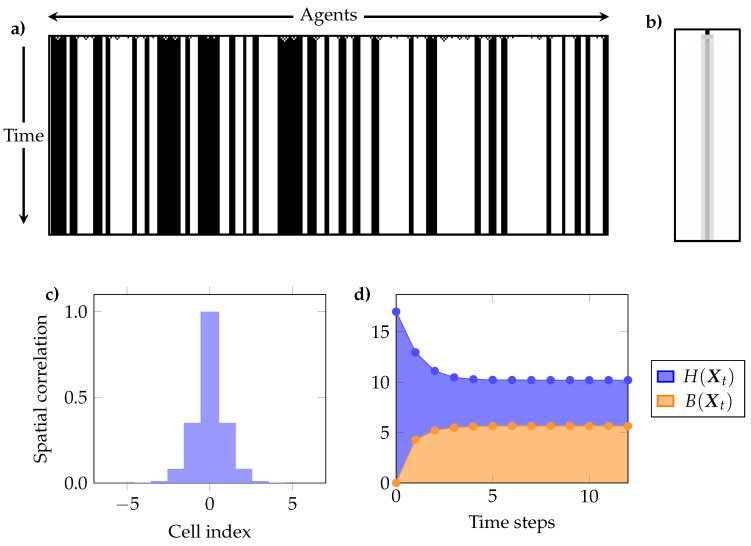
Combined results for rule 232. (**a**) Example of evolution starting from random initial conditions. Note that this example system is larger than the one used in the simulation for plots (**b**–**d**); (**b**) Mutual information between the initial state of a cell and the future state of the same cell and its neighbours (black is higher); (**c**) Profile of pairwise mutual information terms between cells at the pseudo-stationary regime shows a typical exponential decay; (**d**) Time evolution generates interaction reflected by $B(X_{t})$, which is of the same order of magnitude than $R\left(X_{t}\right)=H\left(X_{t}\right)-B\left(X_{t}\right)$. Both *B* and *H* are reported in bits.

**Figure 3:**
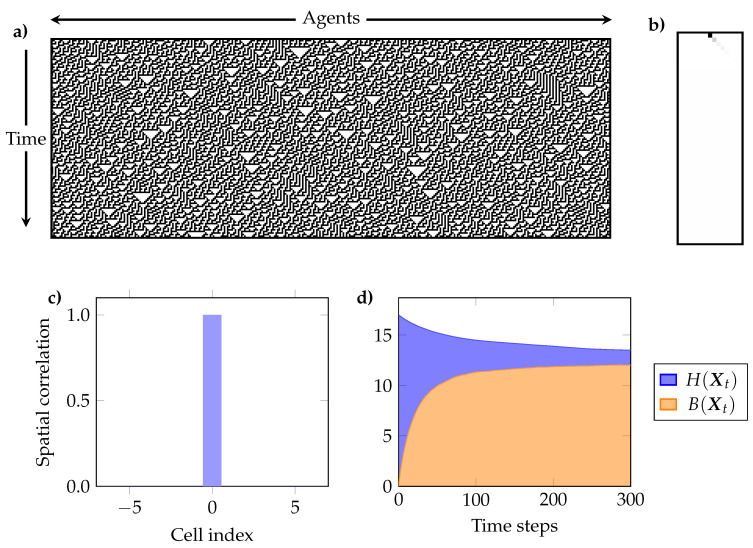
Combined results for rule 30. (**a**) Example of evolution starting from random initial conditions. Note that this example system is larger than the one used in the simulation for plots (**b**–**d**); (**b**) Mutual information between the initial state of a cell and the future state of the same cell and its neighbours (black is higher); (**c**) At the pseudo-stationary regime, there exists no mutual information between any pair of cells; (**d**) Despite having no significant pairwise correlations, the dynamics generate large amounts of interdependency between the cells reflected by a high value of $B(X_{t})$. Both *B* and *H* are reported in bits.

**Figure 4:**
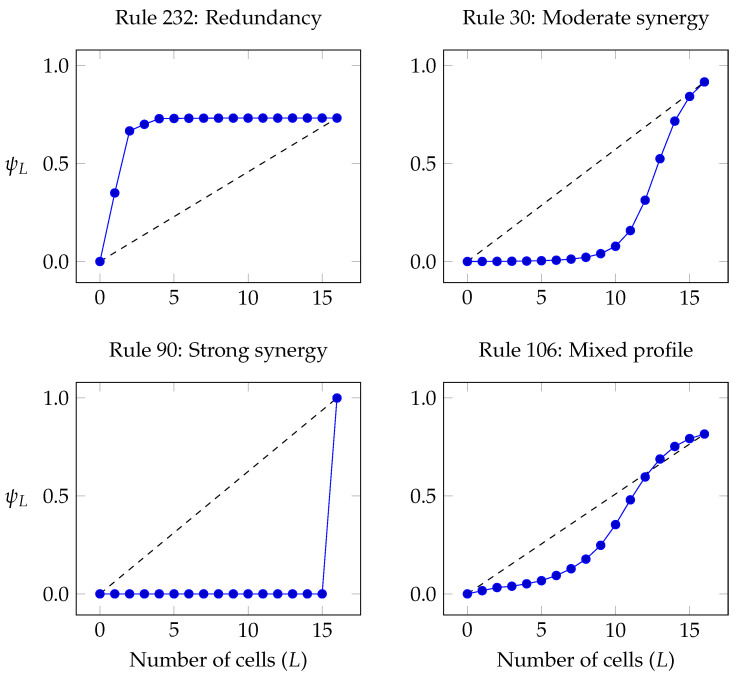
While the concave shape of $\psi_{L}$ for Rule 232 shows that correlations are mostly redundant, the convex shape for Rule 30 shows the dominance of synergies of order 10 or more. Rules 60 and 90 are the only rules that generate purely synergistic structure of the highest order. Results for Rule 106 show an inflection point where $\psi_{L}$ switches from convex to concave, suggesting the coexistence of synergistic small-scale and redundant large-scale structures.

Now we present and discuss the profiles of some well-known rules, which illustrate paradigmatic behaviour. Figure 2, Figure 3 and Figure 4 show results of ECA with N=17 agents, while extended versions of these results for all rules with N=4,…,17 agents can be found in https://cellautomata.xyz. Please note that, as the behaviour of ECA is known to be sometimes affected by the specific number of agents (see e.g., Reference [78]), we only discuss results that are exhibited consistently for the range of studied values of *N*.

#### 6.2.1. Strong Redundancy: Rule 232

Rule 232 is commonly referred to as the majority rule, as one cell’s next state is 1 if and only if two or more of its predecessors are 1. The dynamics of this rule when starting from a random initial condition are governed by interactions between nearest neighbours, which are resolved after few steps into stable configurations (Figure 2a). As a result of this brief interaction, the dynamics generate binding information while decreasing the joint entropy, as shown in Figure 2d.

In agreement with those observations, it is found that one cell at the initial condition has high predictive power over the state of itself and its nearest neighbours in the future (Figure 2b). Correspondingly, the profile of pairwise mutual information terms between cells at the pseudo-stationary regime shows exponentially decaying correlations as a function of cell distance (Figure 2c).

The curve of $\psi_{L}$ shows a concave shape, growing strongly for the first two (nearest) neighbours, growing slightly for the third and fourth nearest neighbours, and remaining then essentially flat (Figure 4). This means that remote neighbours are practically independent, which is consistent with the pairwise correlation profile. Note that knowing all the other cells provides an 75% prediction over a given cell, meaning that there is a non-negligible amount of residual entropy.

In summary, Rule 232 shows the signature of redundancy-dominated self-organisation. This behaviour was found consistently in rules that evolve towards fixed states and rules that evolve towards periodic orbits with relatively short cycle lengths, which are known in the CA literature as Class 1 and Class 2 rules, respectively [73].

#### 6.2.2. Synergistic Profile: Rule 30

Rule 30 is known for generating complex geometric patterns, and has a sensitive dependence to initial conditions [79]. This rule, among others, has provided key insights to understand how simple rules can generate complex structures. For example, similar patterns can be found in the shell of the *conus textile* cone snail species. Rule 30 has also been proposed as a stream cipher for cryptography [80], and has been used as a pseudo-random number generator [81].

Visual inspection suggests that the information processing done by this rule is much more complex than Rule 232. In effect, Figure 3d shows that this rule generates high $B(X_{t})$ through a much longer mixing time. Intriguingly, the predictive information of a single cell seems to disapear after very few steps (Figure 3b), meaning that knowing the state of a single cell of the initial condition is not useful for predicting the state of any cell at later stages. Even more intriguingly, the pseudo-stationary regime shows that each pair of cells is practically independent (Figure 3b), in direct contrast with the high value of $B(X_{t})$.

These aparent paradoxes are solved when one considers high-order correlations by studying the behaviour of $\psi_{L}$ (Figure 4). In effect, the convex shape of the curve shows a pronounced synergistic structure: groups of less than 8 cells show no interdependency, but groups of 15 allow almost perfect prediction! This shows that the self-organisation driven by Rule 30 generates high-order structures. In particular, for arrays of 17 cells, the fact that $\sum_{j=1}^{9}m_{j}\left(X_{t}\right)\approx 0$ implies that the interdependencies are synergies of order 10 or higher.

#### 6.2.3. Pure Synergy: Rules 60 and 90

Rule 90 consists of concatenated xor logic gates: the future state of each cell correponds to the xor of its two precessors. When started from a single active cell, Rule 90 generates a Sierpinsky triangle, while when started from a random initial condition it generates irregular triangular patterns. Rule 90 is known for having connections with number theory, as discussed in Reference [72].

Together with Rule 60, which is also composed by concatenated xors, Rule 90 was found to be the most synergistic rule of all 256 ECA. In fact, for an array of *N* cells started with random initial conditions, after the second step any group of N−1 cells or less is statistically independent. This implies that $\psi_{L}=0$ for all L<N−1, and therefore $m_{L}\left(X_{t}\right)=0$ for all L<N−1 (Figure 4). However, our calculations show that $R(X_{t})=0$ while $B\left(X_{t}\right)=H\left(X_{t}\right)=N-1$, indicating that the binding information of Rules 60 and 90 corresponds exclusively to synergy of the highest order, i.e., $B\left(X_{t}\right)=m_{N-1}\left(X_{t}\right)$.

We found that most ECA rules with attractors of length of the order of the phase space (known as Class 3 and 4 in the CA literature [82]) exhibit synergy-dominated self-organisation. Besides rules 30, 60 and 90 (and the ones in their equivalence classes), rules 18 and 146 have the strongest convexity in their $\psi_{L}$ profiles. Interestingly, the fact that rules 60 and 90 have been found to have the highest synergy is consistent with the crucial role played by xor gates in cryptography (see Reference [61] and Section 4.2).

#### 6.2.4. Coexistence of Convex and Concave Segments in $\psi_{L}$

Interestingly, some rules show both convex and concave sections in $\psi_{L}$. Examples of this phenomenon are rules 14, 22, 41, 54, 62, 73, 106 and 110, with the shape of $\psi_{L}$ being sometimes sensitive to the system size. Rules 106 and 110, in particular, show a clear distinction between a convex segment for small *L* and concave segment for large *L*. When compared with rule 106, rule 110 has its inflection point at a smaller *L*, which could be related with the more localised structures seen in this rule.

Based on these results, we hypothesise that a combination of synergy and redundancy within a single system could provide a richer, or more “complex” structure. However, further investigation in larger systems would be necessary to confirm that the inflection point is actually an intrinsic property of the rule—and not a finite-size effect.

## 7. Discussion

This paper presents an information-theoretic framework to study self-organisation in multi-agent systems, which explores how statistical structures are spontaneously generated by the evolution of coupled dynamical systems. To guarantee the absence of centralised control guiding the process, we restrict ourselves to autonomous systems where each agent can interact directly with only a small number of other agents. To isolate structures that are purely created by the system’s dynamics, we consider the evolution of agents that are initially maximally random and independent.

A fundamental insight behind our framework is the fact that *deterministic dynamical systems are able to create correlations by destroying information*. In effect, we saw that while the temporal evolution of many dynamical systems reduce their joint Shannon entropy, this condition can be the consequence of two qualitatively opposite scenarios: in one case interdependency is created while the stochasticity of each agents is preserved; and in the other mere information dissipation occurs (each agent becomes less random while remaining independent of each other). Following this line of thought, and diverging from the standard literature, we propose to attribute self-organisation to processes where the strength of interdependencies increases with time. In this work we use the binding information as metric of global interdependency strength.

As a second step, we propose a multi-layered description of the attained organisation based on synergies and redundancies of various orders. The key idea is to decompose the information stored in the system, as quantified by the joint Shannon entropy, considering two principles: extension (how many agents are linked), and sharing mode (how many agents need to be measured in order to obtain predictive power). The information sharing mode of order 1 corresponds to redundancy, which takes place when by measuring only one agent one can (partially) predict the state of a number of other agents. Synergy takes place when such predictive power is accessible only when measuring two or more agents simultaneously. We proposed these decompositions as formal structures, without providing an explicit way to compute the values of their components for arbitrary probability distributions. Nevertheless, upper and lower bounds for these components are provided, which in some cases can allow a complete determination of the decomposition.

Using the proposed framework, this work is the first—to the best of our knowledge—to demonstrate cases of high-order statistical synergy in relatively large systems. In particular, we showed that the ECA that corresponds to rule 90 generates maximal synergy, which, according Reference [61] and Section 4.2, could enable the development of interesting cryptographic applications. Moreover, our results suggest that some rules can exhibit a coexistence of redudant and synergistic structures at different scales. However, more work is needed in order to confirm this hypothesis and explore its implications.

Let us remark that our framework does not intend to compare diverse systems on a unidimensional ranking of organisational richness. Accodingly, it would not be correct to claim that rule 90 attains a richer organisation than other ECA. Our framework uses increments in the binding information to detect self-organisation, and then applies an multi-dimensional information decomposition to provide qualitative insight of the result of this process. As a result, different types of structures (e.g., redundant, synergistic or mixed) are acknowledged in their diversity, without trying to collapse their properties into a single number.

An interesting extension of this work would be to use some of the recently proposed measures of synergy (see e.g., [83,84,85,86]) to build exact formulas for the proposed decompositions. This would allow a more precise characterisation of the strength of each information sharing mode. However, this could prove to be challenging, as most of these metrics are designed for systems of three variables with their extensions to larger systems not being straightforward.

Another natural extension would be to apply the presented framework to study continuous coupled dynamical systems, and also their stochastic counterparts (e.g., stochastic differential equations). Interestingly, while the entropy of continous systems can be negative, the binding information is still a non-negative quantity and hence its decomposition can be carried out directly using the framework proposed in Section 4.4. Moreover, all the presented results and methods are valid for systems with random dynamics, with the sole exception of the fact that the joint entropy can increase (in contrast to what was discussed in Section 4.2). The main challenge for this would be to develop faithful estimators of the corresponding densities for cases where analytical expressions are not available. This task could, for example, be approached by using well-established methods of Bayesian inference [87] and density estimation [88].

To study the structure of a particular attractor in a non-ergodic system, one could focus the analysis on the corresponding natural invariant measure (c.f. [89]) instead of studying the evolution from the uniform distribution. It would be of interest to explore if well-known chaotic attractors can be explained in terms of the synergies and redundancies they induce in the corresponding coordinates, which could provide a new link between chaos theory and multivariate information theory. These developments could allow to study real-world phenomena, e.g., sensorimotor control loops [45,90]. Also, this development could enable a bridge between the ideas presented in this paper and the extensive literature of self-organising coupled oscillators (see e.g., [91,92]).

Finally, it is worth emphasising that the statistical character of our proposed framework makes it orthogonal to some well-established self-organisation principles, such as the *enslaving principle* for multi-scale systems [93] or the *free energy principle* for autopoetic organisms [23]. As a matter of fact, it remains to be explored to what extent those principles can be enriched by including multi-layered decompositions in terms of redundancies and synergies. Also, please note that the presented approach to self-organisation is restricted to structures that are generated within known possibilities, which is related to the idea of “weak emergence” [94]. An attractive extension would be to include phenomena related to “strong emergence,” i.e., processes in which evolution can affect the state space itself, generating entirely new configurations for the system to explore. An attractive way of attempting this extension could be to combine the presented framework with the notion of super-exponentially growing phase spaces presented in Reference [95].

## Appendix A. Alternative Approaches to Formalising Global Structure

### Appendix Structure as Geometrical Properties of Attractors

A natural way to attempt a formalisation of structure is by relating it to the notion of *attractor* from the dynamical systems literature. To define what an attractor is, let us note that the set $\left\{x_{s}|x_{s}=\phi_{s}\left(x_{0}\right)\right\}$ corresponds to the *trajectory* that originates from the initial condition $x_{0}\in \Omega$. A set B⊂Ω is stable if it contains the trajectories of all its elements, i.e., for all $y_{0}\in B$ and t∈N we have that $y_{t}=\phi_{t}\left(y_{0}\right)\in B$. An attractor is a set *A* that is stable and has no stable proper (non-empty) subsets. Common attractors are fixed points, limit cycles (i.e., periodic trajectories) and strange attractors [96].

Since the early efforts of Ashby [10,97], it has been noted that self-organisation is a consequence of the tendency of dynamical systems to evolve towards attractors. The system, hence, becomes more “selective” as time passes. Following this rationale, one can argue that the distinctive properties of these attracting configurations are the ones that emerge within the time-evolution.

Could one relate the attractor’s geometrical structure to properties of structure and organisation? An attractive fact is that “interesting” dynamics are usually associated with non-linear equations, which in turn generate strange attractors with exotic geometric properties; while on the other hand the attractors of linear dynamics have uninteresting geometrical structure. Following this line of thought, one could attempt to establish relationships between the geometrical structure of attractors (e.g., in terms of fractal structure) and properties of the organisation attained by the agents. Although this is plausible, the route to develop such endeavour is not straightforward.

### Appendix Structure as Pattern Complexity

Interesting approaches for formalising the concept of structure or pattern can be found in the computer science and signal processing literature. One such approach is to relate pattern strength with the Kolmogorov complexity (KC) [98], which is the length of the shortest computer program that is able to generate the pattern as output. In this way, pattern stength is inversely proportional to the value of the corresponding KC: very structured configurations can be generated by short programs, while random configurations with no structure can only be the output of a program of the same length of the sequence itself.

Using the KC to measure pattern strength is attractive due to its intuitiveness, and because its quantitative nature can allow comparisons between heterogenous structures [99]. Unfortunately, the KC has been proven to be not computable (this imposibility being related to Gödel’s incompletness theorem [100]), which hinders its practical value (practical estimation of the KC can still be attempted via upper bounds, which can be calculated using lossless compression algorithms [99]).

Another weakness of this approach is that the KC does not have, to the best of our knowledge, properties about the relationship between the complexity of a system and the complexity of it parts, nor properties of how the KC evolves in time under diverse dynamical conditions. These two limitations are overcomed by adopting an information-theoretic framework, as we do in the main body of the paper.

## Appendix B. From a Dynamical System to a Stochastic Process

In order to consider probabilities defined over a metric phase space Ω, one needs to introduce a collection of “events,” denoted by B, which correspond to measurable subsets of Ω. It is natural to ask this collection to be a σ-field, so that if $B_{1},B_{2}\in B$ then $B_{1}\cup B_{2}\in B$ and $B_{1}\cap B_{2}\in B$ are guaranteed [101]. A probability measure μ is a function μ:B↦[0,∞) such that μ(Ω)=1, which satisfies the relationship $\mu \left(\cup_{j=1}^{\infty }B_{j}\right)=\sum_{j=1}^{\infty }\mu \left(B_{j}\right)$ if $B_{j}\in B$ for all j∈N and $\cap_{j=1}^{\infty }B_{j}=⌀$. When considering a map $\phi_{t}$ over the phase space, it is natural to require $\phi_{t}$ and B to match together appropiately, i.e., for all B∈B then $\phi_{t}^{-1}\left(B\right)=\left\{x\in \Omega |\phi_{t}\left(x\right)\in B\right\}\in B$. In that way, one can guarantee the consistency of the definition given in (2).

Given a probability distribution $\mu_{0}$, any measurable function Y:Ω↦R can be considered to be a random variable with statistics defined as

(A1) $$P\left\{Y\in I\right\}\mu \left(Y^{-1}\left(I\right)\right)\;.$$

Above, $Y^{-1}\left(I\right)=\left\{x\in \Omega |Y\left(x\right)\in I\right\}$ and I⊂R. Similary, the multivariate stochastic process $X_{t}=\left(X_{t}^{1},\cdots ,X_{t}^{n}\right)$ induced by the map $\phi_{t}$ is defined by the joint statistics are given by

(A2) $$P\left\{X_{t_{0}}^{i_{1}}\in I_{1},\cdots ,X_{t_{m}}^{i_{m}}\in I_{m}\right\}\mu_{0}\left(\cap_{j=1}^{m}\left\{x\in \Omega |\phi_{t_{j}}^{j}\left(x\right)\in I_{j}\right\}\right)\;,$$

where $i_{j}\in \left\{1,2,\cdots ,n\right\}$ are a collection of indices, $t_{1},\cdots ,t_{m}\in T$ is a collection of time points, $I_{j}\subset R$ for j=1,⋯,m, and $\phi_{t}^{j}$ is the *j*-th coordinate of the map at time *t* as defined in Section 3.2. For discrete phase spaces, the joint probability distribution of $X_{t}$ is given by $p_{X_{t}}\left(x\right)=P\left\{X_{t}=x\right\}$ for x∈Ω.

## Appendix C. Information and Entropy

The entropy is a functional over the probability distribution that describes the state of knowledge that an observer has with respect to a given system of interest [28]. In this context, uncertainty in the system corresponds to information that can be potentially extracted by performing adequate measurements.

Following this line of thought, the amout of information needed to specify a single configuration within |Ω| possibilities is log|Ω|, where the base of the logarithm can be choosen according to the preferred units for counting information (bits, nats, or others). If a system with a phase space of cardinality |Ω| at time *t* follows a statistical distribution $p_{X_{t}}$, then this information gets divided as follows [61]:

(A3) $$\log|\Omega |=H\left(X_{t}\right)+N\left(X_{t}\right),$$

where $H\left(X_{t}\right):=-E\left\{\log p_{X_{t}}\left(X_{t}\right)\right\}$ is the joint Shanon entropy of the system, and $N\left(X_{t}\right):=\log\left|\Omega \right|-H\left(X_{t}\right)$ is the “negentropy”. After an observer comes to know the statistics of the system, as encoded by $p_{X_{t}}$, the average amount of information needed to specify a particular configuration decreases from log|Ω| to $H(X_{t})$; therefore, the negentropy corresponds to the bits that are disclosed by the knowledge of the statistics. In contrast, the Shannon entropy measures the information that is not disclosed by the statistics, which can only be obtained when the configuration of the system is actually measured.

## Appendix D. Simulation Details

This appendix describes the procedure for calculating the evolution of probability distributions over ECA (c.f. Section 7). The possible states of an ECA with *N* cells were encoded as a binary numbers, and hence the phase space corresponds to $\Omega =\{0,\cdots ,2^{N}-1\}$. Probability distributions over the phase space were stored as an array $L_{\mu }=\left(\mu \left(0\right),\cdots ,\mu \left(2^{N}-1\right)\right)$, where μ(k)≥0 for all $k\in \{0,\cdots ,2^{N}-1\}$ and $\sum_{k=0}^{2^{N}-1}\mu \left(k\right)=1$.

We considered trajectories over Ω, which correspond to sequences of binary numbers $\left(s_{1},s_{2},\cdots \right)$ such that $s_{k+1}=\phi \left(s_{k}\right)$ with ϕ:Ω→Ω being the function that encondes the ECA rule. Our interest was to find the step at which ϕ(·) brings the trajectory back to a state that has been already visited before. Note that all trajectories end up in a periodic attractor, this being a consequence of the finiteness of Ω. From a trajectory starting at s∈Ω, we store the pair $\left(p_{s},a_{s}\right)$ with $p_{s}$ being the length of the trajectory until reaching a state in the periodic attractor, and $a_{s}$ being the legth of the periodic attractor (i.e., the number of states between the first and the second appearance of a repeated state). The interest of these numbers lays in the fact that

(A4) $$\phi_{t}\left(s\right)=\phi_{K(t,s)}\left(s\right)\;\forall t\in \left\{p_{s},p_{s}+1,\cdots \right\}\;,$$

where $K\left(t,s\right)p_{s}+\left(t-p_{s}\right)\;\mathrm{mod}\;a_{s}$. Above, $\phi_{t}\left(s\right)=\phi \circ \cdots \circ \phi \left(s\right)$ is the *t*-th composition of ϕ with itself.

The ECA is said to have reached a *pseudo-stationary regime* when it has been run for a number of steps $t_{s}$ such that, for any initial distribution $\mu_{0}$, a trajectory of distributions $\left(\mu_{0},\mu_{1},\cdots ,\mu_{t_{s}}\right)$ obtained by time evolution would reach a distribution that has already been visited before. As mentioned in Section 4.1, the set of all probability distributions μ over Ω together with their dynamics form a new dynamical system, which also has periodic attractors. From this point of view, $t_{s}$ is the smallest integer such that all trajectories of distributions reach their periodic attractor. Therefore, the minimal number of steps needed to a reach pseudo-stationary regime, denoted by $t_{0}$, can be calculated as

(A5) $$t_{0}=LCM\left({\left\{a_{s}\right\}}_{s\in \Omega }\right)+\max_{s\in \Omega }p_{s}\;,$$

where LCM stands for the *least common multiple*. Above, the last term ensures that each state entered their periodic attractor, and the former is the smallest number of steps that guarantees a simultaneous full cycle of all the attractors. For the considered ECA with N=17 cells, the largest values found where $t_{0}\approx 10^{14}$.

In order to be able to study the statistics of ECA under pseudo-stationary regimes, we developed an efficient way to compute the evolution of a given initial distribution μ for very large number of steps. Let us represent the initial distribution μ by the array $L_{\mu }$, and the resulting distribution after $t_{0}$ steps as $\mu^{\prime }$ with its corresponding vectorial representation $L_{\mu^{\prime }}$. Our key idea is to compute the trajectory from each s∈Ω only for $K(t_{0},s)$ steps – as additional multiples of $a_{s}$ correspond to mere cycles over its periodic attractor. The general procedure for computing $\mu^{\prime }$ using this idea goes as follows:

1. Initialize the components of $L_{\mu^{\prime }}$ with zeros.
2. For each s∈Ω: compute $s^{\prime }=\phi_{K(t_{0},s)}\left(s\right)$ and then add μ(s) to $\mu^{\prime }\left(s^{\prime }\right)$ (i.e., add $L_{\mu }\left[s\right]$ to $L_{\mu^{\prime }}\left[s^{\prime }\right]$).

This technique resulted to be very efficient, as the largest values found over all ECA rules for N=17 were $\max_{s\in \Omega }p_{s}=1776$ and $\max_{s\in \Omega }a_{s}=78821$.
